# Production performance and survivability of six dual-purpose breeds of chicken under smallholder farmers' management practices in Nigeria

**DOI:** 10.5194/aab-63-387-2020

**Published:** 2020-11-12

**Authors:** Folasade Olubukola Ajayi, Oladeji Bamidele, Waheed Akinola Hassan, Uduak Ogundu, Abdulmojeed Yakubu, Olayinka Olubunmi Alabi, Oludayo Michael Akinsola, Emmanuel Babafunso Sonaiya, Oluwafunmilayo Ayoka Adebambo

**Affiliations:** 1 Department of Animal Science, University of Port-Harcourt, Rivers State, Nigeria; 2 African Chicken Genetic Gains Project National Secretariat, Department of Animal Science, Obafemi Awolowo University, Ile-Ife, Osun State, Nigeria; 3 Department of Animal Science, Usmanu Danfodiyo University, Sokoto, Sokoto State, Nigeria; 4 Department of Animal Science, Federal University of Technology, Owerri, Imo State, Nigeria; 5 Department of Animal Science, Faculty of Agriculture, Nasarawa State University, Keffi, Shabu-Lafia Campus Lafia, Nasarawa State, Nigeria; 6 Department of Animal Science, Landmark University, Omu-Aran, Kwara State, Nigeria; 7 Department of Theriogenology and Production, University of Jos, Jos, Plateau State, Nigeria; 8 Department of Animal Breeding and Genetics, Federal University of Agriculture, Abeokuta, Ogun State, Nigeria

## Abstract

Chickens kept under free-range, backyard or semi-intensive systems in the developing countries have more diverse use and benefits to rural households. Their use varies from region to region and from community to community within a region. The study investigated growth, laying performance and survivability of six improved dual-purpose breeds in five agroecologies of Nigeria represented by the following states: Kebbi (Sudan savanna/northern Guinea savanna); Kwara (northern Guinea savanna/southern Guinea savanna); Nasarawa (southern Guinea savanna/derived savanna); Imo (lowland rainforest/swamp); and Rivers (freshwater swamp/mangrove swamp). On-farm data were obtained from 2100 smallholder poultry farmers that received an average of 30 birds (mixed sexes) of any one of the following dual-purpose breeds (Fulani, FUNAAB Alpha, Kuroiler, Noiler, Sasso and Shika-Brown) randomly allocated to them. The farmers used the backyard, scavenging system of management. Body weight and mortality records for cocks were taken for 18 weeks, while body weight, mortality, egg production and egg weight data were collected for hens up to 72 weeks. Compared with the local cocks (680 g), Kuroiler (1391 g), Sasso (1398 g) and Noiler (1461 g) had over 200 % body weight at 18 weeks. Hen day egg production (HDEP) was higher in Shika-Brown (45.9 %), FUNAAB Alpha (45.8 %) and Kuroiler (45.7 %) compared with the other breeds. Fulani, FUNAAB Alpha and Shika-Brown had higher survivabilities (p<0.05) than Noiler, Sasso and Kuroiler. Ranking of the breeds for growth, laying performance and survivability was as follows: Shika-Brown/Sasso > FUNAAB Alpha/Noiler > Kuroiler > Fulani. The performance of the breeds was significantly (p<0.05) influenced by the agroecologies. The cock body weights for Fulani (1121.1 g), FUNAAB Alpha (1502.4 g) and Noiler (1459.2 g) were highest in Kebbi, while for Kuroiler (1561.0 g), Sasso (1695.9 g) and Shika-Brown (1131.6 g) cock body weights were highest in Imo. Across the states, Imo had the highest HDEP (62.8 %). Overall, the lowland rainforest/ freshwater swamp agroecologies supported the highest production performance of the breeds.

## 1 Introduction

In many developing countries chickens are reared under the free-range, backyard or semi-intensive system as a means of improving the livelihood of the people (Sonaiya, 1990, 2007; Kitalyi, 1998; Guèye, 2000; Kryger et al., 2010; Billah et al., 2013; Yusuf et al., 2014; Alemayehu et al., 2018). A major challenge of smallholder chicken production is the use of local genotypes with a small body size, which offer poor feed quantity and quality resulting in low egg and meat output and high mortality (Yakubu et al., 2007; Mellesse, 2014; Ajayi and Agaviezor, 2016; Sankhyan and Thakur, 2018). A knowledge of the production performance of traits of economic importance is required for formulation of breeding plans aimed at improving the livelihoods of smallholder chicken farmers (Yakubu et al., 2019). Improving genetic potentials of smallholder chicken requires testing different breeds in two or more environments in order to determine the magnitude of genotype × environment interaction (Falconer and Mckay, 1996; Nauta, 2009).

In order to improve the productivity of chicken of smallholder farmers in sub-Saharan Africa, two foreign-sourced tropically adapted breeds from India (Kuroiler) and France (Sasso) and four locally sourced breeds (FUNAAB Alpha, Noiler, Shika-Brown and Fulani) developed in Nigeria (Bamidele et al., 2019) were tested on-farm for growth, egg production and survivability in five agroecological zones in Nigeria. The study was carried out under the African Chicken Genetic Gains (ACGG) project in Nigeria with the aim of guiding decisions on the choice of appropriate smallholder chicken breeds.

## 2 Materials and methods

### 2.1 Description of study area

On-farm data were collected between August 2016 and August 2018 in five different agroecological zones represented by five states: Kebbi (Sudan savanna/northern Guinea savanna), Kwara (northern Guinea savanna/southern Guinea savanna), Nasarawa (southern Guinea savanna/derived savanna), Imo (lowland rainforest/swamp) and Rivers (freshwater swamp/mangrove swamp) states. The climatic conditions of the five agroecologies were as described by Yakubu et al. (2019). Kebbi and Nasarawa had similar average temperature of 28 ${}^{\circ }$C, while average temperatures in Imo, Kwara and Rivers ranged between 26.4 and 26.8 ${}^{\circ }$C. Relative humidity was 47.4 %, 74.0 %, 74.4 %, 80.0 % and 83.4 %, respectively, for Kebbi, Nasarawa, Kwara, Imo and Rivers. Annual rainfall in the five zones also followed the same pattern as the relative humidity. The values are 809, 1169, 1217, 2219 and 2708 mm, respectively, for Kebbi, Nasarawa, Kwara, Imo and Rivers.

In each of the three senatorial districts of each state, two local government areas (LGAs) were randomly selected (i.e. six LGAs) and two villages were randomly selected per LGA giving 12 villages per state and 60 villages in all. A total of 2100 smallholder farmers were randomly selected from the five states at 420 farmers per state. The population of chickens distributed according to breed and agroecological zone (state) is as shown in Table 1.

**Table 1:** The number of chicken breeds distributed based on agroecological zones.

Agroecological	Breed						Total
zone (state)	Sasso	Kuroiler	Shika-	FUNAAB	Noiler	Fulani	
			Brown	Alpha			
Imo	2520	2520	2520	1440	2520	1080	12 600
Kebbi	2520	2520	2520	1440	2520	1080	12 600
Kwara	2520	2520	2520	1440	2520	1080	12 600
Nasarawa	2520	2520	2520	1440	2520	1080	12 600
Rivers	2520	2520	2520	1440	2310	1080	12 390
Total	12 600	12 600	12 600	7200	12 390	5400	62 790

### 2.2 Experimental birds and management

During bird distribution, each of the participating smallholder poultry farmers was allocated an average of 30 pre-vaccinated 6-weeks-old growers of any one of the six breeds while ensuring that all the breeds were represented in each village. Each farmer, selected by a simple random sampling technique, in each of the villages received randomly selected birds of any one of the previously allocated breeds. The birds were managed under free range with basic shelter and feed supplementation provided according to each farmer's ability. Locally available supplementary feeds used by the farmers included kitchen waste, agricultural by-products and plant parts. These feeds were mostly energy-based feed resources with a similar nutrient composition across the five agroecologies (Oyewale et al., 2020). Farmers were trained during community innovation platforms on best management practices for improved health and productivity of birds. Newcastle disease vaccination and a deworming service were provided through community animal health workers (CAHWs) that were trained, supplied and supervised by veterinary officers. The cocks were raised to 20 weeks old for meat purpose, while hens were raised for eggs up to 72 weeks. At 20 weeks, the farmers were free to slaughter the cocks for meat consumption or to sell them for income, while eggs produced by the hens, over the 52-week laying period, served as a source of nutrition and income (Alabi et al., 2020). This study was approved by the International Livestock Research Institute (ILRI) Institutional Research Ethics Committee (IREC) with reference no. ILRI-IREC2015-08/1. All applicable veterinary permits for the importation, use and testing of the imported breeds, solely for research purposes, were obtained (Bamidele et al., 2019). Each farmer gave written informed consent to participate in the study.

### 2.3 Research hypothesis

#### 2.3.1 Null hypothesis

There is no significant difference in growth performance, egg production and survivability of the six chicken breeds in the five agroecological zones of Nigeria

#### 2.3.2 Alternative hypothesis

The growth performance, egg production and survivability of the six chicken breeds are significantly different in the five agroecological zones under study in Nigeria.

### 2.4 Data collection and statistical analyses

Data were collected using the Open Data Kit (ODK) preloaded onto a Lenovo tablet (TAB 2 A7-30H). A field officer was assigned to each village to collect data for body weight and mortality every 4 weeks (28 d) from 6 to 72 weeks. In order to reduce the stress on the birds, data collection at the households started 1–2 d after bird distribution, but this inadvertently resulted in mortality due to non-genetic factors (theft, predation and stress). Farmers were pre-informed prior to field officers' visits; all birds were weighed during morning hours after overnight fasting using a suspended weighing scale with a sensitivity of 100 g. Mortality, egg production and egg weight records were taken every 2 weeks (14 d) from 22 to 72 weeks. All collected data were uploaded to the ILRI data server directly from the village. All raw data are available as open-access data at http://data.ilri.org/portal/dataset/acggonfarmng (last access: 17 April 2018).

Growth rate and egg production performance data were analysed using unbalanced type-III two-way analysis of variance (ANOVA) implemented in the R car (version 3.0-2) package (Fox and Weisberg, 2011) to test the effect of breed, agroecologies and their interactions on the production performance of birds. Significant differences were separated using a Tukey test (α=0.05) for multiple comparisons through R least square means (version 2.30-0) (Length, 2016) and R multcomp (version 1.4-10) (Hothorn et al., 2008) packages.

The Cox proportional hazard regression analysis using R survival (version 2.42-3) (Therneau, 2015) and survminer (version 0.4.4) (Kassambara and Kosinski, 2019) packages was also used to investigate the effect of breed and agroecologies on the survival of birds. The significance of these factors was tested using Kaplan–Meier and log-rank tests. Hazard ratios were derived from Cox models. Proportional hazards assumed a non-significant relationship between scaled Schoenfeld residuals and time. All statistical analyses were performed in R version 3.5.1 (R Core Team, 2018).

## 3 Results

### 3.1 Growth performance of six breeds of chicken

Significant breed variations were observed in body weight and body weight gains of male and female birds tested on-farm (Tables 2 and 3). Fulani (303.93±10.87 g) and Shika-Brown (361.08±16.38 g) had the lowest body weights at 6 weeks. The highest coefficient of variation (CV) was recorded for FUNAAB Alpha (12.97 %) and Shika-Brown (11.11 %), respectively. Breed, as a factor, significantly influenced the growth rate of male birds from 6 to 18 weeks old. Noiler males showed superiority in growth over the other five breeds from 6 to 14 weeks as shown in Table 2. However, at 18 weeks, the body weight of Noiler (1461.28±63.15 g), Kuroiler (1390.82±33.82 g) and Sasso (1398.77±32.39 g) were not statistically different (p>0.05) from one another. Fulani had the lowest body weight (813.75 g) at 18 weeks.

The CV among the male birds was also highest in FUNAAB Alpha (11.05 %) and Noiler (10.59 %), while Sasso (5.68 %) and Kuroiler (5.97 %) had the lowest values at 18 weeks. The foreign-sourced breeds (Kuroiler and Sasso) had the lowest CV with similar body weights at 18 weeks compared with the other four locally sourced breeds (Noiler, Fulani, FUNAAB Alpha and Shika-Brown) that were developed in Nigeria.

In the females across the six breeds (Table 3), body weights at 6 weeks were lower than for their male counterparts. The differences in body weights of males with respect to their female counterparts at 6 weeks were as follows: Fulani (25.22 g), FUNAAB Alpha (32.19 g), Kuroiler (81.8 g), Noiler (71.31 g), Sasso (56.19 g) and Shika-Brown (36.79 g). At 18 weeks, male birds were 111.21 g (Fulani), 209.21 g (FUNAAB Alpha), 174.13 g (Kuroiler), 131.06 g (Noiler), 148.83 g (Sasso) and 125.44 g (Shika-Brown) heavier than their female counterparts. There was no statistical difference (p>0.05) in body weights of female birds of Noiler, Kuroiler and Sasso from 6 to 18 weeks of age.

The CV in female body weights at 6 weeks ranking from highest to lowest is FUNAAB Alpha (21.59 %), Shika-Brown (11.38 %), Fulani (10.44 %), Kuroiler (5.16 %), Sasso (4.49 %) and Noiler (3.77 %). However, at 18 weeks old, Noiler had the highest CV (12.77 %) compared with Kuroiler (6.18 %) and Sasso (6.93 %).

**Table 2:** Body weight (g) of male birds of the six breeds tested in ACGG-NG (NG – Nigeria) project zones (6–18 weeks) (2016–2017).

Age	Breed																				
(weeks)	N	Fulani	CV	N	FUNAAB Alpha	CV	N	Kuroiler	CV	N	Noiler	CV	N	Sasso	CV	N	Shika-Brown	CV	N	Mean	CV
6	1532	303.93 ± 10.87${}^{f}$	8.76	2371	442.98 ± 23.45${}^{d}$	12.97	4603	616.79 ± 16.12${}^{b}$	6.4	4106	729.77 ± 10.51${}^{a}$	3.53	4641	565.08 ± 11.23${}^{c}$	4.87	5600	361.08 ± 16.38${}^{e}$	11.11	22 853	503.27 ± 66.23	32.24
10	1218	462.56 ± 17.63${}^{e}$	9.34	2136	691.07 ± 24.177${}^{c}$	8.57	3718	838.91 ± 16.46${}^{b}$	4.81	3251	909.87 ± 16.09${}^{a}$	4.33	3826	817.37 ± 17.55${}^{b}$	5.26	4916	517.83 ± 14.70${}^{d}$	6.95	19 065	706.27 ± 74.50	25.84
14	1140	670.94 ± 23.66${}^{d}$	8.64	1795	938.17 ± 31.10${}^{c}$	8.12	3400	1141.29 ± 25.88${}^{b}$	5.55	2676	1314.39 ± 52.97${}^{a}$	9.87	3406	1019.51 ± 24.29${}^{b}$	5.84	4214	728.11 ± 21.59${}^{d}$	7.26	16 631	968.74 ± 99.89	25.26
18	1016	813.75 ± 29.61${}^{d}$	8.91	1581	1202.63 ± 54.27${}^{b}$	11.05	2623	1390.82 ± 33.82${}^{a}$	5.97	2038	1461.28 ± 63.15${}^{a}$	10.59	2778	1398 ± 32.39${}^{a}$	5.68	3658	978.63 ± 36.16${}^{c}$	9.05	13 694	1169.42 ± 122.11	4.63

Values are least squares means ± standard error; means within rows sharing no common superscript were significantly different (P<0.05); N: number of samples; CV %: coefficient of variation.

**Table 3:** Body weight (g) of female birds of the six breeds tested in ACGG-NG project zones (6–18 weeks) (2016–2017).

Age	Breed																				
(weeks)	N	Fulani	CV	N	FUNAAB Alpha	CV	N	Kuroiler	CV	N	Noiler	CV	N	Sasso	CV	N	Shika-Brown	CV	N	Mean	CV
6	2168	278.71 ± 11.88${}^{d}$	10.44	3217	475.17 ± 41.89${}^{c}$	21.59	5318	534.99 ± 11.26${}^{b}$	5.16	4127	658.46 ± 10.13${}^{a}$	3.77	7082	508.89 ± 9.33${}^{\mathrm{bc}}$	4.49	6299	324.29 ± 15.06${}^{d}$	11.38	28 211	463.42 ± 57.40	30.34
10	1796	395.89 ± 14..35${}^{e}$	8.79	2886	607.12 ± 25.42${}^{c}$	10.26	4241	761.89 ± 22.07${}^{\mathrm{ab}}$	7.1	3321	792.86 ± 14.70${}^{a}$	4.54	5877	463.24 ± 14.85${}^{b}$	7.85	5428	463.235 ± 14.85${}^{d}$	7.85	23 549	580.71 ± 68.38	28.84
14	1718	583.24 ± 22.39${}^{d}$	9.4	2549	789.11 ± 20.44${}^{c}$	6.34	3756	1012.66 ± 20.98${}^{b}$	5.07	2837	1236.22 ± 60.43${}^{a}$	11.97	5026	954.80 ± 21.82${}^{b}$	5.6	4698	627.71 ± 18.58${}^{d}$	7.25	20 584	867.29 ± 101.50	28.67
18	1494	702.54 ± 25.15${}^{d}$	8.78	2242	993.42 ± 32.20${}^{b}$	7.94	3033	1216.69 ± 30.72${}^{a}$	6.18	2433	1330.22 ± 69.34${}^{a}$	12.77	4162	1249.17 ± 35.32${}^{a}$	6.93	4137	853.19 ± 31.12${}^{c}$	8.93	17 501	1057.54 ± 101.37	23.48

Values are least squares means ± standard error; means within rows sharing no common superscript were significantly different (P<0.05); N: number of samples; CV %: coefficient of variation.

### 3.2 Effect of agroecological zones on the body weight of male birds

Body weight of male birds varied significantly (p<0.05) at 6 weeks in the five agroecologies where the six breeds were tested (Table 4). Body weight of male birds at 6 weeks was highest for Sasso (858.05±23.69 g) in Imo, Noiler in Kebbi (737.42±16.10 g), Kuroiler in Kwara (848.06±24.25 g), and for Noiler in Nasarawa (791.52±19.51 g) and Rivers (591.17±24.74 g). FUNAAB Alpha had the lowest 6-week body weight in Imo (246.32±31.34 g), and Shika-Brown had the lowest in Kebbi (298.55±16.10 g), Nasarawa (240.46±23.83 g) and Rivers (240.74±23.52 g), while Fulani had the lowest body weight in Kwara (259.06±37.31 g). The trend in body weight increase of male birds at 10 and 14 weeks old was consistent with what was recorded at 6 weeks for all the six breeds across the five agroecologies (Table 4). The CV was highest for Fulani at all ages (6–18 weeks) for male birds in all the five agroecologies. The values ranged from 5.11 % in Imo at 6 weeks to 7.03 % in Rivers at 18 weeks.

The body weight of male birds at 18 weeks in Imo for Sasso was 1695.81 g, while the lowest body weight was recorded in Fulani (794.83 g). In Kebbi, Kuroiler had the highest body weight and Shika-Brown the lowest. In Kwara, the highest body weight was in Kuroiler and lowest in Fulani. In both Nasarawa and Rivers, the highest body weight was in Noiler and the lowest in Fulani.

**Table 4:** Effect of agroecology on body weights (g) of male birds of the six breeds tested in ACGG-NG (NG – Nigeria) project zones (6–18 weeks) (2016–2017). Data in bold font, i.e. N/mean, in the table represents the number of birds/mean body weights according to breeds and age in the five different agroecological zones in Nigeria.

Age	Breed	N	Imo	CV	N	Kebbi	CV	N	Kwara	CV	N	Nasarawa	CV	N	Rivers	CV	N	Mean	CV
(weeks)																			
6	Fulani	350	315.90 ± 36.19${}^{\mathrm{de}}$	5.11	336	374.73 ± 24.30${}^{c}$	2.89	218	259.06 ± 37.31${}^{d}$	6.43	332	307.23 ± 35.23${}^{\mathrm{cd}}$	5.12	278	298.49 ± 36.74${}^{\mathrm{cd}}$	5.49	1514	311.08 ± 18.65	2.68
6	FUNAAB Alpha	350	246.32 ± 31.34${}^{e}$	5.68	464	548.86 ± 21.27${}^{b}$	1.73	512	570.65 ± 32.31${}^{b}$	2.53	569	356.57 ± 31.86${}^{\mathrm{bc}}$	3.99	502	306.87 ± 30.92${}^{\mathrm{cd}}$	4.5	2397	405.85 ± 65.30	7.18
6	Kuroiler	1096	606.06 ± 23.69${}^{c}$	1.75	834	580.61 ± 16.41${}^{b}$	1.26	1091	848.06 ± 24.25${}^{a}$	1.28	822	429.70 ± 23.83${}^{b}$	2.48	848	401.01 ± 23.66${}^{\mathrm{bc}}$	2.63	4691	573.09 ± 79.66	6.21
6	Noiler	892	724.21 ± 22.74${}^{b}$	1.4	812	737.42 ± 16.10${}^{a}$	0.97	873	788.64 ± 23.14${}^{a}$	1.31	835	791.52 ± 19.51${}^{a}$	1.1	795	591.17 ± 24.74${}^{a}$	1.87	4207	726.59 ± 36.42	2.24
6	Sasso	1022	858.05 ± 23.69${}^{a}$	1.23	860	541.31 ± 16.41${}^{b}$	1.35	1030	441.39 ± 24.43${}^{c}$	2.47	860	451.24 ± 23.25${}^{b}$	2.3	896	452.44 ± 23.38${}^{b}$	2.31	4668	548.89 ± 79.38	6.46
6	Shika-Brown	1332	408.19 ± 23.69${}^{d}$	2.59	1223	298.55 ± 16.10${}^{c}$	2.41	949	296.11 ± 24.25${}^{d}$	3.66	1021	240.46 ± 23.83${}^{d}$	4.42	1020	240.74 ± 23.52${}^{d}$	4.36	5545	296.81 ± 30.60	4.6
**6**	N**/mean**	**5042**	**526.46** ± **98.69**	**8.37**	**4529**	**513.58** ± **63.8**	**5.55**	**4673**	**533.99** ± **100.93**	**8.44**	**4439**	**429.45** ± **79.06**	**8.22**	**4339**	**381.79** ± **52.17**	**6.1**	**23 022**		**0**
10	Fulani	322	454.00 ± 52.97${}^{d}$	5.21	314	630.23 ± 31.21${}^{c}$	2.21	194	404.29 ± 40.57${}^{d}$	4.48	172	392.18 ± 51.83${}^{d}$	5.9	216	391.43 ± 57.21${}^{\mathrm{cd}}$	6.52	1218	454.43 ± 45.42	4.46
10	FUNAAB Alpha	282	561.30 ± 45.88${}^{\mathrm{cd}}$	3.65	436	845.07 ± 27.03${}^{b}$	1.43	460	790.03 ± 36.38${}^{b}$	2.06	494	498.27 ± 45.63${}^{\mathrm{cd}}$	4.09	464	478.66 ± 47.24${}^{\mathrm{bcd}}$	4.41	2136	634.67 ± 76.40	5.37
10	Kuroiler	993	1031.48 ± 34.68${}^{b}$	1.5	735	800.93 ± 20.94${}^{b}$	1.17	934	933.62 ± 28.31${}^{a}$	1.35	412	615.37 ± 32.47${}^{\mathrm{bc}}$	2.36	652	565.36 ± 35.25${}^{\mathrm{bc}}$	3.05	3726	789.35 ± 89.45	5.06
10	Noiler	673	933.90 ± 34.68${}^{b}$	1.66	591	1008.15 ± 20.94${}^{a}$	0.93	701	857.76 ± 27.25${}^{\mathrm{ab}}$	1.42	791	936.24 ± 32.47${}^{a}$	1.55	495	755.36 ± 38.57${}^{a}$	2.28	3251	898.28 ± 42.92	2.13
10	Sasso	931	1185.75 ± 34.93${}^{a}$	1.31	779	747.66 ± 20.68${}^{b}$	1.23	897	600.23 ± 28.12${}^{c}$	2.09	420	705.27 ± 32.47${}^{b}$	2.06	799	585.92 ± 35.25${}^{b}$	2.69	3826	764.97 ± 109.57	6.39
10	Shika-Brown	1280	644.67 ± 34.93${}^{c}$	2.42	1058	488.82 ± 20.68${}^{d}$	1.89	775	420.53 ± 28.88${}^{d}$	3.07	912	411.07 ± 34.49${}^{d}$	3.75	891	359.96 ± 34.60${}^{d}$	4.29	4916	465.01 ± 49.38	4.74
**10**	N**/mean**	**4481**	**801.85** ± **118.48**	**6.6**	**3913**	**753.48** ± **73.2**	**4.34**	**3961**	**667.74** ± **92.53**	**6.19**	**3201**	**593.07** ± **84.32**	**6.35**	**3517**	**522.78** ± **59.33**	**5.07**	**19 073**		**0**
14	Fulani	316	666.59 ± 76.64${}^{c}$	5.13	290	890.23 ± 56.39${}^{c}$	2.83	177	506.33 ± 52.67${}^{d}$	4.64	149	627.88 ± 71.98${}^{c}$	5.12	208	617.72 ± 81.02${}^{\mathrm{cd}}$	5.86	1140	661.75 ± 63.03	4.25
14	FUNAAB Alpha	280	880.33 ± 68.09${}^{c}$	3.45	311	1124.09 ± 50.43${}^{b}$	2	407	816.68 ± 46.60${}^{\mathrm{bc}}$	2.55	429	845.33 ± 67.75${}^{\mathrm{bc}}$	3.58	391	737.30 ± 70.17${}^{\mathrm{bcd}}$	4.25	1818	880.75 ± 65.25	3.31
14	Kuroiler	985	1320.13 ± 50.17${}^{\mathrm{ab}}$	1.7	558	1147.15 ± 38.31${}^{b}$	1.49	857	1073.62 ± 35.12${}^{a}$	1.46	369	884.22 ± 44.81${}^{b}$	2.26	623	934.64 ± 54.48${}^{b}$	2.6	3392	1071.95 ± 77.89	3.24
14	Noiler	568	1276.87 ± 50.17${}^{b}$	1.75	417	1326.09 ± 39.33${}^{a}$	1.32	626	967.76 ± 36.25${}^{\mathrm{ab}}$	1.67	668	1157.34 ± 45.67${}^{a}$	1.76	397	1179.80 ± 59.59${}^{a}$	2.25	2676	1181.57 ± 61.75	2.33
14	Sasso	919	1513.14 ± 50.53${}^{a}$	1.49	652	1103.55 ± 37.36${}^{b}$	1.511	784	813.49 ± 35.33${}^{c}$	1.94	361	939.37 ± 46.28${}^{b}$	2.2	690	868.36 ± 54.85${}^{\mathrm{bc}}$	2.82	3406	1047.58 ± 126.20	5.38
14	Shika-Brown	1216	901.32 ± 51.28${}^{c}$	2.54	723	671.79 ± 38.81${}^{d}$	2.58	700	583.63 ± 34.90${}^{d}$	2.67	807	607.52 ± 51.32${}^{c}$	3.77	768	519.59 ± 54.48${}^{d}$	4.68	4214	656.77 ± 65.81	4.47
**14**	N**/mean**	**4284**	**1093.06** ± **132.38**	**5.41**	**2951**	**1043.82** ± **93.52**	**3.99**	**3551**	**793.59** ± **88.75**	**4.99**	**2783**	**843.61** ± **83.97**	**4.44**	**3077**	**809.57** ± **97**	**5.35**	**16 646**		**0**
18	Fulani	314	794.83 ± 94.51${}^{c}$	5.31	268	1120.08 ± 76.17${}^{b}$	3.04	158	592.14 ± 57.97${}^{c}$	4.37	138	694.31 ± 77.88${}^{d}$	5.01	150	730.16 ± 114.93${}^{b}$	7.03	1028	786.30 ± 89.66	5.09
18	FUNAAB Alpha	272	1072.33 ± 83.97${}^{\mathrm{bc}}$	3.5	279	1502.35 ± 66.96${}^{a}$	1.99	404	886.10 ± 49.64${}^{b}$	2.5	341	1109.14 ± 77.88${}^{\mathrm{bc}}$	3.13	331	883.36 ± 98.18${}^{\mathrm{ab}}$	4.96	1627	1090.66 ± 112.91	4.62
18	Kuroiler	805	1561.00 ± 66.28${}^{a}$	1.9	464	1514.77 ± 54.67${}^{a}$	1.61	718	1201.17 ± 36.77${}^{a}$	1.37	286	1145.30 ± 52.28${}^{b}$	2.04	450	1199.77 ± 82.03${}^{a}$	3.05	2723	1324.40 ± 88.04	2.97
18	Noiler	423	1457.01 ± 64.21${}^{a}$	1.97	192	1459.24 ± 63.45${}^{a}$	1.94	432	1087.53 ± 39.24${}^{a}$	1.61	575	1417.81 ± 55.95${}^{a}$	1.76	356	1220.31 ± 84.46${}^{a}$	3.09	1978	1328.38 ± 74.62	2.51
18	Sasso	836	1695.81 ± 64.21${}^{a}$	1.69	447	1494.36 ± 54.67${}^{a}$	1.63	677	879.90 ± 38.20${}^{b}$	1.94	317	1314.79 ± 52.65${}^{\mathrm{ab}}$	1.79	521	1160.75 ± 78.42${}^{a}$	3.02	2798	1309.12 ± 139.66	4.76
18	Shika-Brown	1180	1131.62 ± 63.24${}^{b}$	2.49	596	886.71 ± 54.26${}^{b}$	2.73	595	725.18 ± 38.71${}^{\mathrm{bc}}$	2.38	618	855.40 ± 57.35${}^{\mathrm{cd}}$	2.99	612	749.79 ± 77.10${}^{b}$	4.59	3601	869.74 ± 72.24	3.71
**18**	N**/mean**	**3830**	**1285.43** ± **139.47**	**4.84**	**2246**	**1329.59** ± **107.72**	**3.62**	**2984**	**895.34** ± **91.59**	**4.57**	**2275**	**1089.46** ± **111.51**	**4.57**	**2420**	**990.69** ± **93.59**	**4.22**	**13 755**		**0**

Values are least square means ± standard error; means within columns sharing no common superscript were significantly different (P<0.05); N: number of samples; CV %: coefficient of variation.

### 3.3 Effect of agroecology on the body weight of female birds

In the females (Table 5), across the agroecologies, Noiler was significantly (p<0.05) heavier than all the other breeds at 6 weeks, except in Imo where Sasso (697.31 g) was heavier. At 18 weeks, the highest body weight observed for each breed across the agroecologies was as follows: Fulani – 952.76 g; FUNAAB Alpha – 1294.52 g (Kebbi); Noiler – 1365.39 g (Nasarawa); Kuroiler – 1464.87 g; Sasso – 1489.72 g; and Shika-Brown – 961.46 g (Imo). The breeds with the highest (p<0.05) female body weight within the agroecologies were Kuroiler (1464.87 g) and Sasso (1489.72 g) in Imo, FUNAAB Alpha (1294.52 g), Sasso (1298.02 g), Kuroiler (1298.24 g) and Noiler (1329.47 g) in Kebbi, Kuroiler (1119.54 g) in Kwara, Sasso (1320.52 g) and Noiler (1365.39 g) in Nasarawa, and Noiler (1173.11 g) in Rivers. At 6 weeks, Kuroiler had the lowest CV at Imo (1.57 %), Kebbi (2.63 %) and Kwara (5.8 %), while Fulani (0.98 %) and Noiler (10.75 %) had the lowest CV at Nasarawa and Rivers, respectively. Also, it was observed that Shika-Brown (Kwara, 23.33 %; Rivers, 25.59 %), FUNAAB Alpha (Nasarawa, 11.24 %; Imo, 50.02 %) and Fulani (Kebbi, 8.63 %) had the highest CV. From 14 to 18 weeks, Fulani had the highest CV in all five agroecological zones with values that ranged between 13.78 % (Kebbi) and 31.24 % (Rivers) at 14 weeks and 15.50 % (Kwara) and 37.79 % (Rivers) at 18 weeks.

The effect of the five agroecologies on body weights of female birds of the six breeds was also studied during the laying period from 22 to 70 weeks (Table 6). Female birds showed a significant statistical difference (p<0.05) in body weights in Imo, Kebbi, Kwara, Nasarawa and Rivers in the six breeds during the laying period.

The difference in body weight between the highest (Kebbi) and the lowest (Kwara) at 26 and 30 weeks was 588.48 and 586.29 g, respectively. This pattern of weight difference was consistent for the two zones up to 48 weeks. At 54 weeks, Nasarawa had the lowest body weight (1418.32±38.35 g) with a difference of 523.13 g from the highest body weight recorded in Kebbi. The body weights of female birds were not significantly different (p<0.05) in Imo, Kebbi and Rivers from 50 to 70 weeks old (Table 6), but birds in Nasarawa maintained the lowest body weight up to 70 weeks. The CV was relatively low across all the five agroecological zones for all the breeds tested. The values ranged between 4.71 % in Imo at 30 weeks to 7.51 % in Kwara at 70 weeks old.

**Table 5:** Effect of agroecology on body weights (g) of female birds of the six breeds tested in ACGG-NG project zones (6–18 weeks) (2016–2017).

Age	Breed	N	Imo	CV	N	Kebbi	CV	N	Kwara	CV	N	Nasarawa	CV	N	Rivers	CV	N	Mean	CV
(weeks)																			
6	Fulani	465	281.45 ± 24.56${}^{\mathrm{cd}}$	3.89	520	335.62 ± 19.32${}^{c}$	8.63	342	353.66 ± 67.17${}^{c}$	8.48	388	230.82 ± 5.06${}^{d}$	0.98	453	248.85 ± 37.59${}^{\mathrm{cd}}$	16.78	2168	290.08 ± 23.88	9.96
6	FUNAAB Alpha	441	231.07 ± 21.27${}^{d}$	50.02	722	499.78 ± 16.67${}^{b}$	3.33	721	651.45 ± 57.96${}^{\mathrm{ab}}$	8.9	572	348.05 ± 39.14${}^{c}$	11.24	761	287.81 ± 31.17${}^{\mathrm{cd}}$	15.59	3217	403.63 ± 76.47	22.93
6	Kuroiler	1009	512.57 ± 16.08${}^{b}$	1.57	1247	485.98 ± 12.78${}^{b}$	2.63	1038	756.35 ± 43.88${}^{a}$	5.8	876	430.94 ± 31.56${}^{b}$	3.66	1148	367.91 ± 23.85${}^{\mathrm{bc}}$	12.97	5318	510.75 ± 66.22	31.37
6	Noiler	880	638.29 ± 15.23${}^{a}$	5.34	863	702.41 ± 12.47${}^{a}$	3.98	860	751.82 ± 43.88${}^{a}$	13.08	738	638.97 ± 17.48${}^{a}$	6.13	786	557.78 ± 24.77${}^{a}$	10.75	4127	657.85 ± 32.84	12.08
6	Sasso	1503	697.31 ± 15.97${}^{a}$	5.13	1370	466.30 ± 12.70${}^{b}$	6.1	1467	573.15 ± 43.88${}^{\mathrm{bc}}$	17.14	1103	437.63 ± 31.64${}^{b}$	16.1	1639	404.19 ± 23.56${}^{b}$	13.06	7082	515.72 ± 53.50	23.23
6	Shika-Brown	1181	356.48 ± 16.31c	10.25	1307	272.59 ± 12.47${}^{c}$	4.57	1554	421.26 ± 43.88${}^{c}$	23.33	1062	214.25 ± 8.22${}^{d}$	8.59	1195	229.71 ± 24.29${}^{d}$	25.59	6299	298.86 ± 39.33	31.84
**6**	N**/mean**	**5479**	**452.86** ± **78.65**	**38.9**	**6029**	**460.45** ± **61.01**	**29.68**	**5982**	**584.62** ± **68.81**	**26.36**	**4739**	**383.44** ± **64.20**	**37.5**	**5982**	**349.38** ± **49.99**	**34.63**	**28 211**		**0**
10	Fulani	428	379.65 ± 41.13${}^{d}$	24.26	477	557.42 ± 39.87${}^{\mathrm{cd}}$	16.02	292	384.77 ± 33.13${}^{d}$	19.28	275	318.01 ± 17.71${}^{d}$	13.48	347	346.08 ± 48.82${}^{\mathrm{cd}}$	34.14	1819	397.17 ± 41.84	25.49
10	FUNAAB Alpha	396	501.46 ± 35.62${}^{\mathrm{cd}}$	15.91	677	731.06 ± 34.53${}^{b}$	10.58	637	602.75 ± 29.46${}^{c}$	10.94	545	518.28 ± 29.17${}^{\mathrm{bc}}$	12.61	631	392.32 ± 41.64${}^{\mathrm{bcd}}$	25.69	2886	549.17 ± 56.47	24.88
10	Kuroiler	972	895.05 ± 26.93${}^{b}$	7.28	1066	729.21 ± 26.42${}^{b}$	8.77	805	879.68 ± 21.87${}^{a}$	6.02	504	610.15 ± 24.03${}^{\mathrm{ab}}$	9.53	894	477.41 ± 31.07${}^{\mathrm{bc}}$	15.75	4241	718.30 ± 79.73	26.86
10	Noiler	713	858.37 ± 27.12${}^{b}$	7.64	674	889.78 ± 26.74${}^{a}$	7.27	693	755.95 ± 21.60${}^{b}$	6.91	700	772.71 ± 23.36${}^{a}$	7.32	541	702.88 ± 32.78${}^{a}$	11.29	3321	795.94 ± 34.28	10.42
10	Sasso	1353	1013.04 ± 27.32${}^{a}$	6.53	1146	683.62 ± 26.26${}^{\mathrm{bc}}$	9.29	1282	532.00 ± 22.01${}^{c}$	10.12	681	632.53 ± 22.86${}^{\mathrm{ab}}$	8.75	1415	520.97 ± 30.50${}^{b}$	14.17	5877	676.43 ± 89.53	32.03
10	Shika-Brown	1128	573.36 ± 27.12${}^{c}$	11.45	1098	430.82 ± 26.58${}^{d}$	14.93	1201	354.43 ± 22.45${}^{d}$	15.32	897	426.41 ± 50.40${}^{\mathrm{cd}}$	28.6	1104	315.47 ± 31.07${}^{d}$	23.83	5428	420.10 ± 44.10	20.99
**10**	N**/mean**	**4990**	**703.49** ± **103.14**	**35.48**	**5138**	**670.32** ± **64.71**	**23.36**	**4910**	**584.93** ± **84.15**	**34.81**	**3602**	**546.35** ± **65.84**	**29.16**	**4932**	**459.19** ± **58.15**	**30.75**	**23 572**		**0**
14	Fulani	421	594.77 ± 65.46${}^{c}$	26.63	450	774.45 ± 44.10${}^{c}$	13.78	265	454.96 ± 46.25${}^{c}$	24.6	235	562.41 ± 57.15${}^{c}$	24.59	324	507.35 ± 65.51${}^{d}$	31.24	1695	578.79 ± 54.43	22.75
14	FUNAAB Alpha	363	771.87 ± 56.69${}^{c}$	17.77	545	990.62 ± 38.47${}^{\mathrm{ab}}$	9.39	556	684.75 ± 39.61${}^{b}$	13.84	508	820.93 ± 37.94${}^{\mathrm{bc}}$	11.18	577	571.52 ± 59.81${}^{\mathrm{cd}}$	25.32	2549	767.94 ± 69.97	22
14	Kuroiler	924	1210.01 ± 42.86${}^{\mathrm{ab}}$	8.57	858	1009.28 ± 29.36${}^{\mathrm{ab}}$	7	701	996.75 ± 30.28${}^{a}$	7.35	441	874.35 ± 45.39${}^{\mathrm{bc}}$	12.56	832	833.53 ± 45.36${}^{b}$	13.17	3756	984.78 ± 65.78	16.16
14	Noiler	663	1085.59 ± 42.55${}^{b}$	9.49	529	1109.99 ± 29.93${}^{a}$	6.53	606	930.56 ± 29.70${}^{a}$	7.72	605	1210.73 ± 21.71${}^{a}$	4.34	434	1059.98 ± 48.83${}^{a}$	11.11	2837	1079.37 ± 45.14	10.12
14	Sasso	1292	1285.53 ± 43.17${}^{a}$	8.13	908	971.20 ± 28.81${}^{b}$	7.18	1116	695.22 ± 29.89${}^{b}$	10.4	580	946.06 ± 47.72${}^{\mathrm{ab}}$	12.2	1130	755.00 ± 44.75${}^{\mathrm{bc}}$	14.34	5026	930.60 ± 103.47	26.9
14	Shika-Brown	1074	787.39 ± 43.48${}^{c}$	13.36	827	579.67 ± 29.74${}^{d}$	12.41	1085	508.70 ± 29.70${}^{c}$	14.13	788	580.18 ± 34.39${}^{c}$	14.38	924	434.03 ± 45.68${}^{d}$	25.47	4698	577.99 ± 58.90	24.66
**14**	N**/mean**	**4737**	**955.86** ± **112.95**	**28.59**	**4117**	**905.87** ± **79.05**	**21.12**	**4329**	**711.82** ± **88.93**	**30.23**	**3157**	**832.44** ± **99.07**	**28.8**	**4221**	**693.57** ± **95.67**	**33.38**	**20 561**		**0**
18	Fulani	296	668.97 ± 79.98${}^{d}$	28.93	293	952.76 ± 66.05${}^{b}$	16.77	163	505.32 ± 49.92${}^{e}$	23.91	85	722.21 ± 46.28${}^{c}$	15.5	191	572.85 ± 89.47${}^{c}$	37.79	1028	684.42 ± 76.88	27.18
18	FUNAAB Alpha	295	934.57 ± 70.14${}^{\mathrm{cd}}$	18.16	496	1294.52 ± 57.61${}^{a}$	10.77	487	774.69 ± 45.16c${}^{d}$	14.11	481	1001.91 ± 45.06${}^{\mathrm{ab}}$	10.88	424	734.57 ± 78.16${}^{\mathrm{bc}}$	25.74	2183	948.05 ± 99.69	25.44
18	Kuroiler	781	1464.87 ± 52.36${}^{a}$	8.64	671	1298.24 ± 43.42${}^{a}$	8	650	1119.54 ± 33.49${}^{a}$	7.24	294	1059.36 ± 50.27${}^{\mathrm{ab}}$	11.48	592	949.74 ± 62.72${}^{\mathrm{ab}}$	15.98	2988	1178.35 ± 91.20	18.73
18	Noiler	564	1224.03 ± 50.92${}^{b}$	10.01	373	1329.47 ± 47.74${}^{a}$	8.69	466	955.12 ± 34.82${}^{b}$	8.82	591	1365.39 ± 30.94${}^{a}$	5.48	394	1173.11 ± 64.97${}^{a}$	13.4	2388	1209.42 ± 72.44	14.49
18	Sasso	1134	1489.72 ± 53.52${}^{a}$	8.69	698	1298.02 ± 44.53${}^{a}$	8.3	953	773.88 ± 34.36${}^{c}$	10.74	393	1320.52 ± 78.58${}^{a}$	14.4	878	924.48 ± 61.19${}^{\mathrm{ab}}$	16.02	4056	1161.32 ± 133.81	27.88
18	Shika-Brown	1053	961.46 ± 53.12${}^{c}$	13.37	693	774.67 ± 45.73${}^{b}$	14.27	937	620.42 ± 33.49d${}^{e}$	13.06	678	826.81 ± 66.31b${}^{c}$	19.4	777	655.93 ± 60.22${}^{c}$	22.22	4138	767.86 ± 61.35	19.33
**18**	N**/mean**	**4123**	**1123.94** ± **132.83**	**28.6**	**3224**	**1157.95** ± **95.98**	**20.06**	**3656**	**791.5** ± **90.58**	**27.69**	**2522**	**1049.37** ± **105.25**	**24.27**	**3256**	**835.11** ± **90.62**	**26.26**	**16 781**		**0**

Values are least square means ± standard error; means within columns sharing no common superscript were significantly different (P<0.05); N: number of samples; CV%: coefficient of variation.

**Table 6:** Effect of agroecology on body weights (g) of female birds of the six breeds during laying in ACGG-NG project zones (22–70 weeks) (2016–2018).

Age	N	Imo	CV	N	Kebbi	CV	N	Kwara	CV	N	Nasarawa	CV	N	Rivers	CV	N	Mean	CV
(weeks)																		
22	4214	1336.46 ± 30.22${}^{b}$	5.06	2662	1397.04 ± 30.70${}^{b}$	4.91	3121	929.43 ± 29.80${}^{d}$	7.17	2292	1541.41 ± 30.88${}^{a}$	4.48	2745	1148.02 ± 33.05${}^{c}$	6.44	15 034	1270.47 ± 106.09	18.67
26	3930	1482.82 ± 32.10${}^{b}$	4.84	2052	1634.82 ± 34.95${}^{a}$	4.78	2665	1046.34 ± 32.10${}^{c}$	6.86	2054	1471.18 ± 32.48${}^{b}$	4.94	2395	1376.78 ± 35.76${}^{b}$	5.81	13 096	1402.39 ± 98.15	15.65
30	3664	1539.72 ± 32.45${}^{b}$	4.71	1657	1718.83 ± 37.43${}^{a}$	4.87	2404	1132.54 ± 32.99${}^{c}$	6.51	1985	1457.37 ± 32.21${}^{b}$	4.94	2111	1465.79 ± 36.78${}^{b}$	5.61	11 821	1462.85 ± 95.02	14.52
34	3514	1616.45 ± 34.40${}^{b}$	4.76	1202	1843.48 ± 42.20${}^{a}$	5.12	2076	1207.48 ± 35.18${}^{d}$	6.52	1877	1476.17 ± 33.68${}^{c}$	5.1	1953	1616.88 ± 38.66${}^{b}$	5.35	10 622	1552.09 ± 104.35	15.03
38	3295	1662.37 ± 35.04${}^{b}$	4.71	820	1883.70 ± 45.90${}^{a}$	5.45	1555	1304.49 ± 36.71${}^{d}$	6.29	1713	1464.49 ± 34.65${}^{c}$	5.29	1768	1650.06 ± 39.43${}^{b}$	5.34	9151	1593.02 ± 98.06	13.76
42	3014	1702.43 ± 35.33${}^{b}$	4.64	685	1947.25 ± 48.57${}^{a}$	5.58	1548	1421.32 ± 39.22${}^{c}$	6.17	1532	1487.89 ± 35.28${}^{c}$	5.3	1566	1689.73 ± 40.80${}^{b}$	5.4	8345	1649.72 ± 92.54	12.54
46	2867	1734.79 ± 37.70${}^{b}$	4.86	595	1927.84 ± 55.68${}^{a}$	6.46	1391	1459.82 ± 42.67${}^{c}$	6.54	1505	1486.03 ± 37.39${}^{c}$	5.63	1356	1745.96 ± 44.00${}^{\mathrm{ab}}$	5.64	7714	1670.89 ± 87.88	11.76
50	2603	1761.27 ± 39.01${}^{a}$	4.95	460	1937.05 ± 57.76${}^{a}$	6.67	1260	1500.9 ± 44.60${}^{b}$	6.64	1353	1524.28 ± 37.59${}^{b}$	5.51	1319	1787.63 ± 45.55${}^{a}$	5.7	6995	1702.23 ± 83.10	10.92
54	2409	1789.63 ± 40.08${}^{a}$	5.01	430	1941.45 ± 61.79${}^{a}$	7.11	1111	1543.74 ± 46.65${}^{b}$	6.75	1050	1418.32 ± 38.35${}^{b}$	6.05	1173	1788.05 ± 46.42${}^{a}$	5.82	6173	1696.24 ± 94.28	12.43
58	2158	1788.05 ± 41.60${}^{a}$	5.2	292	1950.11 ± 73.65${}^{a}$	8.44	972	1551.48 ± 51.00${}^{b}$	7.35	1308	1369.10 ± 39.03${}^{c}$	6.37	1035	1856.37 ± 48.26${}^{a}$	5.81	5765	1703.02 ± 106.36	13.97
62	1994	1796.98 ± 42.08${}^{\mathrm{ab}}$	5.24	246	1926.95 ± 75.82${}^{a}$	8.8	893	1632.52 ± 52.27${}^{b}$	7.16	1160	1440.12 ± 39.14${}^{c}$	6.08	787	1906.75 ± 50.15${}^{a}$	5.88	5080	1740.66 ± 91.51	11.76
66	1934	1798.71 ± 44.29${}^{\mathrm{ab}}$	5.51	234	1905.92 ± 83.11${}^{\mathrm{ab}}$	9.75	857	1659.45 ± 55.45${}^{\mathrm{bc}}$	7.47	995	1496.40 ± 40.82${}^{c}$	6.1	779	1948.15 ± 52.49${}^{a}$	6.02	4799	1761.73 ± 83.00	10.53
70	1665	1843.64 ± 46.08${}^{\mathrm{ab}}$	5.59	180	2086.98 ± 88.72${}^{a}$	9.51	763	1712.49 ± 57.51${}^{b}$	7.51	1225	1386.18 ± 40.02${}^{c}$	6.46	551	1957.54 ± 53.58${}^{a}$	6.12	4384	1797.37 ± 119.99	14.93

Values are least squares means ± standard error; means within rows sharing no common superscript were significantly different (P<0.05); N: number of samples; CV %: coefficient of variation.

### 3.4 Egg production performance

Egg production characteristics of the six breeds in the five agroecological zones are shown in Table 7. Mortality for all the breeds was lowest in Imo resulting in a higher total egg number (223 379 eggs) and mean hen day production (HDEP) (62.84 %) in the 52-week laying period, compared to the other states. Although Kebbi (2972) had a higher total number of birds at 52 weeks than Imo (2465), the total egg number in 52 weeks was 192 731 eggs higher in Imo than Kebbi. This difference may be attributed to the high temperature prevalent in Kebbi. Kwara had the lowest survival of birds at 72 weeks (613 birds) and the lowest mean HDEP (23.18 %) during the laying period. The total egg number in Nasarawa (81 397) was higher than Rivers (76 948); however, the mean HDEP was higher in Rivers (57.40 %) than in Nasarawa (33.50 %). It is not known whether pilferage or poor records is responsible for these anomalies. Egg production performance of the six breeds across agroecologies revealed that Shika-Brown had the highest population of birds at 72 weeks and HDEP of 45.92 %. FUNAAB Alpha and Kuroiler were next in mean HDEP at 45.78 % and 45.68 %, respectively. Across the agroecologies, Fulani and Noiler had the lowest (43.02 g) and the highest (55.31 g) egg weights, while the mean egg weight was highest in Kwara (57.49 g) and lowest in Nasarawa (47.99 g).

**Table 7:** Total egg production per breed and by location in ACGG Nigeria project zones (2016–2018).

State	Breed	No. birds at	No. birds at	Total no.	Average egg	HDEP (%)
		22 weeks	72 weeks	of eggs in	weight (g)	
				52 weeks		
Imo	Fulani	399	195	14 046	38.57	62.04
	FUNAAB Alpha	331	186	14 228	49.29	60.81
	Kuroiler	822	469	37 131	56.09	65.46
	Noiler	607	364	34 978	55.17	62.59
	Sasso	1210	575	33 852	54.99	61.26
	Shika-Brown	1057	676	89 144	53.50	64.87
	Total	4426	2465	223 379	51.27	62.84
Kebbi	Fulani	433	296	2857	41.43	40.85
	FUNAAB Alpha	542	354	5222	56.07	46.02
	Kuroiler	900	616	4110	55.81	48.45
	Noiler	526	394	5393	58.36	32.51
	Sasso	945	646	2681	54.41	43.83
	Shika-Brown	971	666	10 385	53.78	36.49
	Total	4317	2972	30 648	53.31	41.36
Kwara	Fulani	253	84	2134	46.87	17.63
	FUNAAB Alpha	501	34	1791	56.54	20.00
	Kuroiler	638	24	4248	63.94	24.69
	Noiler	482	163	8382	61.19	32.74
	Sasso	960	165	3839	61.13	19.76
	Shika-Brown	978	143	6001	55.25	24.25
	Total	3812	613	26 395	57.49	23.18
Nasarawa	Fulani	253	142	4829	44.43	33.66
	FUNAAB Alpha	539	317	12 431	48.36	33.16
	Kuroiler	512	248	9051	49.06	35.35
	Noiler	872	765	21 423	48.53	27.37
	Sasso	685	316	9312	50.68	35.97
	Shika-Brown	840	530	24 351	46.95	35.47
	Total	3701	2318	81 397	47.99	33.50
Rivers	Fulani	363	98	9655	43.78	55.91
	FUNAAB Alpha	551	187	15 082	49.68	68.89
	Kuroiler	663	199	10 794	52.05	54.44
	Noiler	308	91	14 238	53.31	52.32
	Sasso	1009	357	6866	50.89	44.31
	Shika-Brown	871	463	20 313	49.35	68.53
	Total	3765	1395	76 948	49.84	57.40
Across agro-	Fulani	1701	815	33 521	43.02	42.02
ecologies	FUNAAB Alpha	2464	1078	48 754	51.98	45.78
	Kuroiler	3535	1556	65 334	55.39	45.68
	Noiler	2795	1777	84 414	55.31	41.51
	Sasso	4809	2059	56 550	54.42	41.03
	Shika-Brown	4717	2478	150 194	51.77	45.92
	Total	20 021	9763	438 767	51.98	43.66

HDEP: mean hen day egg production.

### 3.5 Bird mortality at growing and laying phase

**Figure 1:**
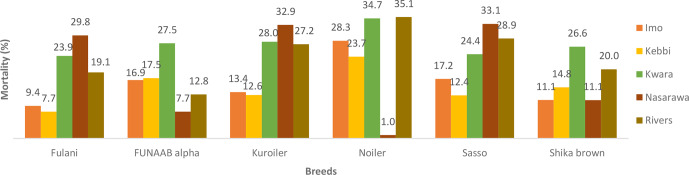
Actual mortality of male birds during growing phase in ACGG project zones (6–18 weeks) (2016–2017).

**Figure 2:**
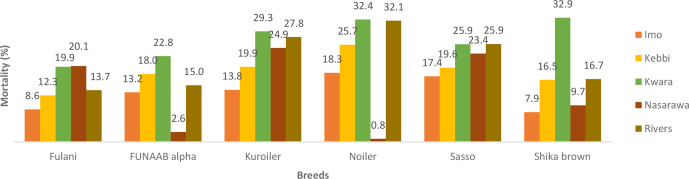
Actual mortality of female birds during growing phase in ACGG project zones (6–18 weeks) (2016–2017).

**Figure 3:**
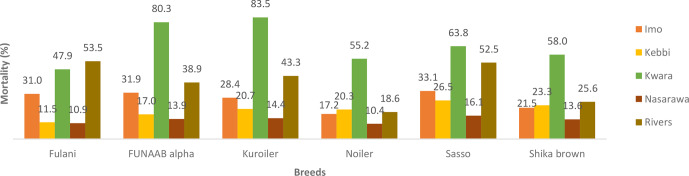
Actual mortality of female birds during laying phase in ACGG project zones (20–72 weeks) (2016–2018).

Breed and agroecologies influenced the mortality rates in male and female birds during the growing phase (Figs. 1 and 2). Nasarawa had the highest mortality rates for Fulani male (29.8 %) and female birds (20.1 %). Kwara had the highest mortality for both male and female birds of FUNAAB Alpha and Shika-Brown and only female birds of Noiler (32.4 %), Kuroiler (29.3 %) and Sasso (25.9 %). Rivers recorded the highest mortality for male Noiler (35.1 %). During the laying phase, Kwara had the highest mortality rate for all the breeds, except for Fulani, which had the highest mortality rate in Rivers (Fig. 3).

### 3.6 Survival and risk factors associated with breeds of bird and agroecologies

#### 3.6.1 Growing phase (6–18 weeks)

**Figure 4:**
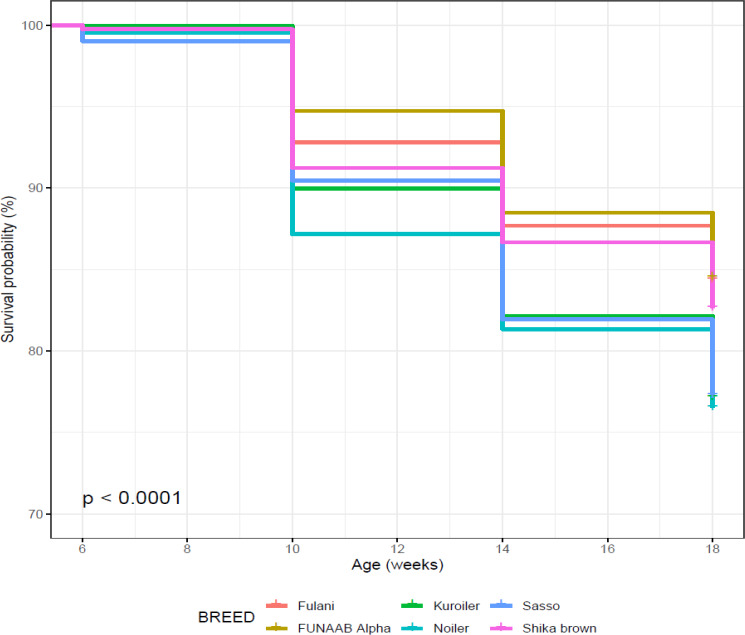
Effect of breed on overall on-farm survival performance of birds (male and female) during growing phase in ACGG Nigeria project zones (6–18 weeks) (2016–2017).

**Figure 5:**
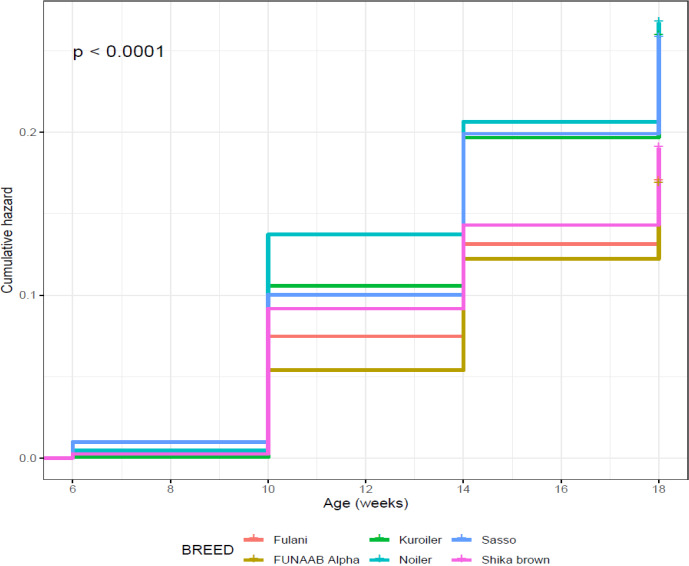
Effect of breed on overall on-farm cumulative hazard of birds (male and female) during growing phase in ACGG Nigeria project zones (6–18 weeks) (2016–2017).

Using age in weeks as survival time and initial and final number of birds and breeds as the covariates, the four breeds developed in Nigeria (FUNAAB Alpha, Fulani, Shika-Brown and Noiler) had higher probabilities of survival (Table 8) compared to the two foreign breeds. Kuroiler and Sasso had survival values of 0.772 ± 0.005 and 0.773 ± 0.005 and cumulative hazard ratios of 0.259 ± 0.005 and 0.258 ± 0.005, respectively from 6 to 18 weeks. The Cox proportional hazard regression model shows that Sasso had the highest risk between 6 and 10 weeks and Noiler between 10 and 18 weeks (Fig. 4), while FUNAAB Alpha maintained the lowest risk from 10 to 18 weeks (Fig. 5).

**Figure 6:**
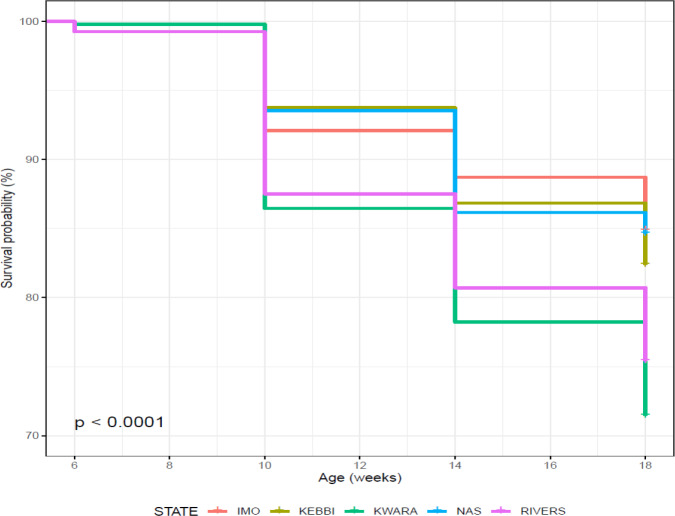
Effect of agroecology on overall on-farm survival performance of birds (male and female) during growing phase in ACGG Nigeria project zones (6–18 weeks) (2016–2017).

**Figure 7:**
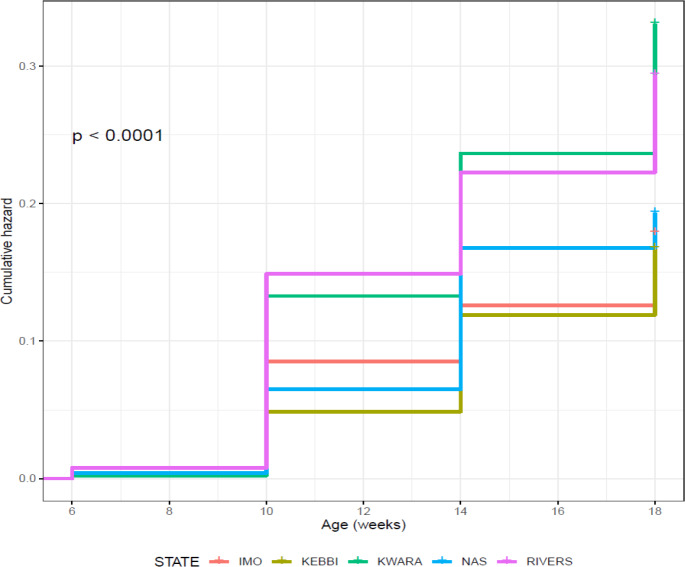
Effect of agroecologies on overall on-farm cumulative hazard of birds (male and female) during growing phase in ACGG Nigeria project zones (6–18 weeks) (2016–2017).

Overall survival probabilities of males and females during the growing phase (6–18 weeks) within agroecologies are shown in Table 9. Imo had the highest survival probability (0.849 ± 0.004) for all birds, which was followed closely by Nasarawa and Kebbi. Overall survival probability for all breeds was slightly higher in Rivers (0.754 ± 0.006) than Kwara (0.715 ± 0.006). Kaplan–Meier survival curves show fewer probabilities of survival in Kwara and Rivers from 6 to 18 weeks (Fig. 6) and a cumulative force of mortality of 0.336 ± 0.006 (Table 8). Significant cumulative hazards were recorded for the overall performance of birds (Fig. 7) during the growing stage (6–18 weeks). A Cox regression model revealed that Rivers had more birds at risk of death from 6 to 14 weeks, while between 14 and 18 weeks old Kwara had more birds at risk of death (Fig. 7).

**Table 8:** Effect of breed on overall on-farm survival performance of birds (male and female) during growing phase in ACGG Nigeria project zones (6–18 weeks) (2016–2017).

Breeds	IN	FN	Survival probability ± SE	Cumulative hazard ± SE	Log rank (P value)
Fulani	3682	3229	0.844 ± 0.007	0.17 ± 0.007	2.27 $\times 10^{-69}$
FUNAAB Alpha	5614	4968	0.845 ± 0.006	0.168 ± 0.006	
Kuroiler	10 009	8221	0.772 ± 0.005	0.259 ± 0.005	
Noiler	8329	6775	0.765 ± 0.006	0.267 ± 0.006	
Sasso	11 750	9628	0.773 ± 0.005	0.258 ± 0.005	
Shika-Brown	11 844	10 265	0.827 ± 0.004	0.19 ± 0.004	

IN and FN: initial and final number of birds; SE: standard error; log rank: test of homogeneity for differences in survival.

**Table 9:** Effect of agroecology on overall on-farm survival performance of birds (male and female) during growing phase in ACGG Nigeria project zones (6–18 weeks) (2016–2017).

State	IN	FN	Survival probability ± SE	Cumulative hazard ± SE	Log rank (P value)
Imo	10 351	9183	0.849 ± 0.004	0.164 ± 0.004	1.92 $\times 10^{-201}$
Kebbi	10 438	9065	0.824 ± 0.005	0.194 ± 0.005	
Kwara	10 550	8253	0.715 ± 0.006	0.336 ± 0.006	
Nasarawa	9747	8399	0.846 ± 0.004	0.167 ± 0.004	
Rivers	10 142	8186	0.754 ± 0.006	0.283 ± 0.006	

IN and FN: initial and final number of birds; SE: standard error; log rank: test of homogeneity for differences in survival.

#### 3.6.2 Laying phase (20–72 weeks)

**Figure 8:**
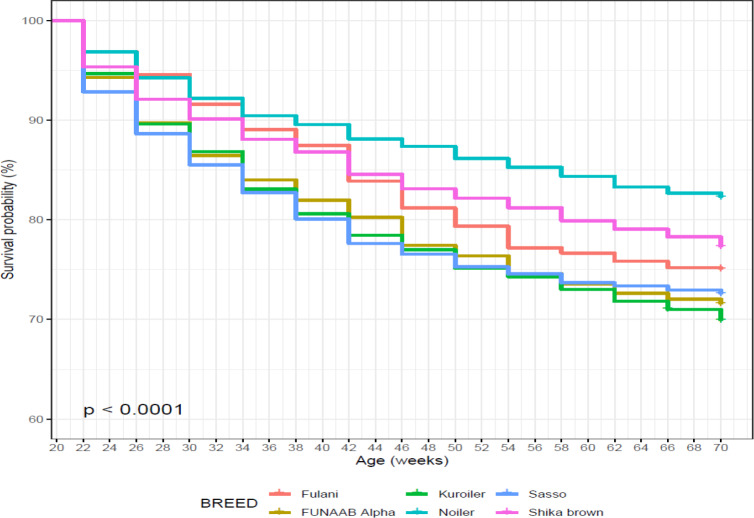
Effect of breed on survival performance of female birds raised on-farm in ACGG Nigeria project zones (20–72 weeks) (2016–2018).

**Figure 9:**
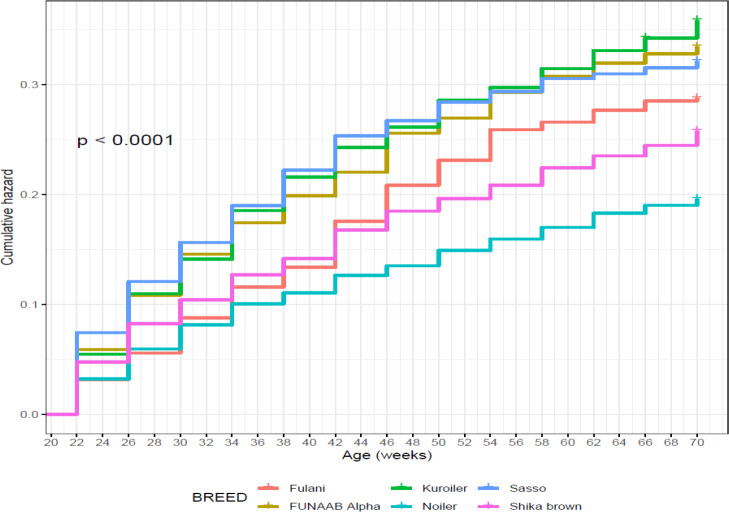
Effect of breed on cumulative hazard of female birds raised on-farm during laying phase in ACGG Nigeria project zones (20–72 weeks) (2016–2018).

Survival probability was influenced significantly by breed of birds during the laying period (Table 10). Noiler had the highest survivability (0.822) and the lowest number of birds at risk of death (0.196), while Kuroiler was the lowest in survival ability (0.699), having more birds at risk of death. Survival curves also showed that Noiler had more female birds during laying than other breeds (Fig. 8), and the cumulative hazard (Fig. 9) for birds at risk of death was highest in Kuroiler laying hens.

**Figure 10:**
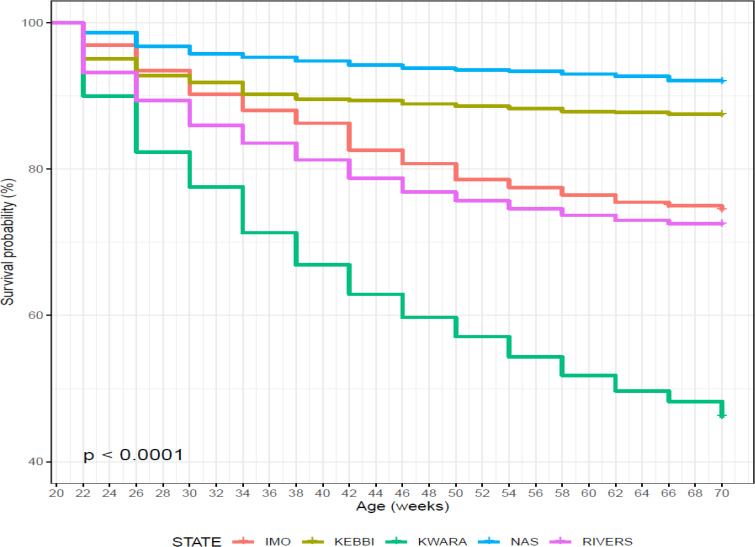
Effect of agroecology on survival performance of female birds raised on-farm in ACGG Nigeria project zones (20–72 weeks) (2016–2018).

**Figure 11:**
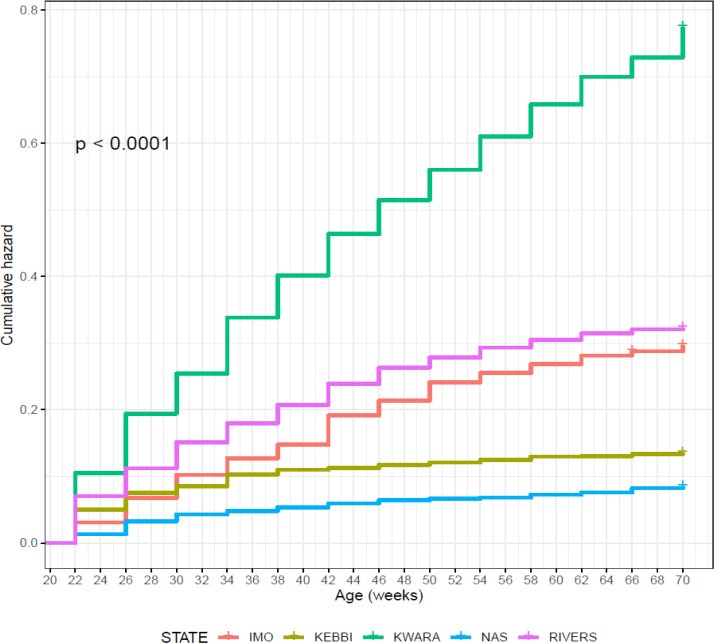
Effect of agroecology on cumulative hazard of female birds raised on-farm in ACGG Nigeria project zones (20–72 weeks) (2016–2018).

Birds in Nasarawa had the highest survivability potential of 91.9 % and the lowest risk of death (Table 11), while birds in Kwara had the lowest survivability (46.1 %) and the highest risk of death (0.775). Survival and cumulative hazard for agroecologies are shown in Figs. 10 and 11.

**Table 10:** Effect of breed on survival performance of female birds raised on-farm during laying phase in ACGG Nigeria project zones (20–72 weeks) (2016–2018).

Breeds	IN	FN	Survival probability ± SE	Cumulative hazard ± SE	Log rank (P value)
Fulani	1701	1279	0.75 ± 0.014	0.287 ± 0.014	3.87 × 10${}^{-35}$
FUNAAB Alpha	2464	1775	0.716 ± 0.013	0.335 ± 0.013	
Kuroiler	3535	1906	0.699 ± 0.011	0.358 ± 0.011	
Noiler	2795	2311	0.822 ± 0.009	0.196 ± 0.009	
Sasso	4809	3508	0.725 ± 0.009	0.321 ± 0.009	
Shika-Brown	4717	3693	0.773 ± 0.008	0.258 ± 0.008	

IN and FN: initial and final number of birds; SE: standard error; log rank: test of homogeneity for differences in survival.

**Table 11:** Effect of agroecologies on survival performance of female birds raised on-farm in ACGG Nigeria project zones (22–70 weeks) (2016–2018).

State	IN	FN	Survival probability ± SE	Cumulative hazard ± SE	Log rank (P value)
Imo	4426	2715	0.744 ± 0.009	0.296 ± 0.009	0
Kebbi	4317	3778	0.874 ± 0.006	0.135 ± 0.006	
Kwara	3812	1839	0.461 ± 0.018	0.775 ± 0.018	
Nasarawa	3701	3408	0.919 ± 0.005	0.085 ± 0.005	
Rivers	3765	2732	0.724 ± 0.01	0.323 ± 0.01	

IN and FN: initial and final number of birds SE: standard error; log rank: test of homogeneity for differences in survival.

### 3.7 Breed × environment interaction on survival and risk factors of birds

#### 3.7.1 Growing phase (6–18 weeks)

**Figure 12:**
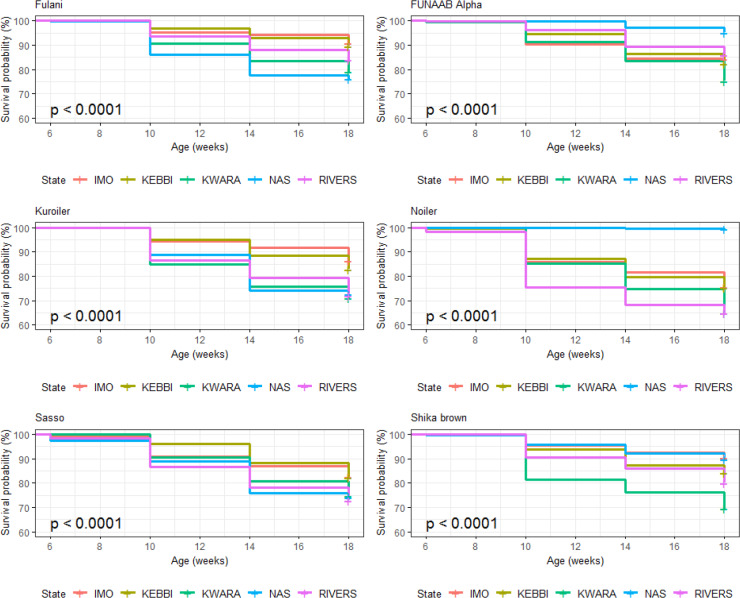
Breed by environment interaction on overall survivability of birds (breeds) during growing phase in ACGG Nigeria project zones (6–18 weeks) (2016–2017).

**Figure 13:**
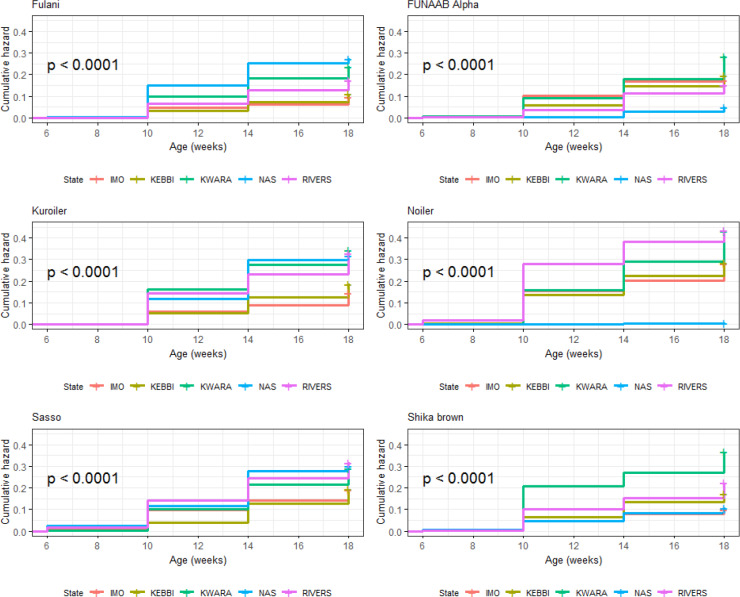
Breed by environment interaction on cumulative hazard of birds (breeds) during growing phase in ACGG Nigeria project zones (6–18 weeks) (2016–2017).

**Figure 14:**
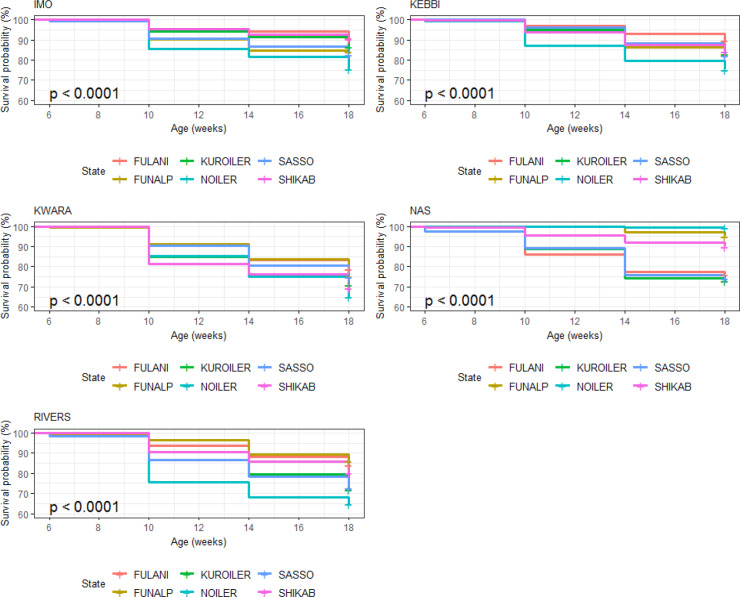
Breed by environment interaction on overall survivability of birds (agroecologies) during growing phase in ACGG Nigeria project zones (6–18 weeks) (2016–2017).

**Figure 15:**
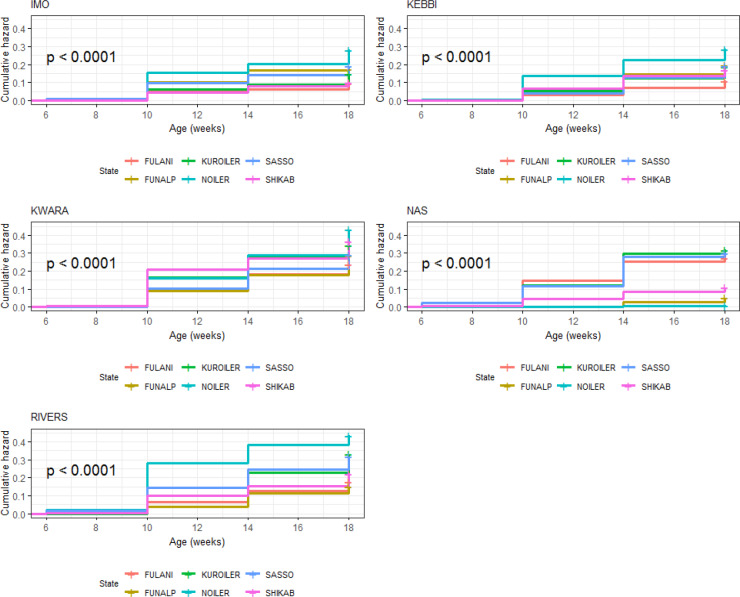
Breed by environment interaction on overall cumulative hazard of birds (agroecologies) during growing phase in ACGG Nigeria project zones (6–18 weeks) (2016–2017).

Breed by environment interaction effect on the growth of birds revealed that two breeds in Imo that survived best were Shika-Brown (90.4 %) and Fulani (90.8 %) (Table 12). In Kebbi, Fulani (89.6 %) and Shika-Brown (84.3 %) and in Kwara Fulani (78.9 %) and FUNAAB Alpha (75.2 %) had the highest survival probabilities. In Nasarawa, the highest survival probabilities were recorded for Noiler (99.2 %) and FUNAAB Alpha (95 %); and in Rivers survival probabilities were highest for FUNAAB Alpha (85.9 %) and Fulani (83.9 %). Survival probabilities of growing birds according to age and breeds are displayed in Fig. 12. Breeds with the highest risk of death were Fulani (at 14–18 weeks) in Nasarawa, Noiler (at 10–18 weeks) in Rivers and Shika-Brown (at 10–18 weeks) in Kwara (Fig. 13). Agroecology by breed interaction varied with respect to probabilities of survival and cumulative hazards across the five zones at different ages of the birds (Figs. 14 and 15). Noiler had its highest risk of death in Imo, Kebbi and Rivers (Fig. 15).

**Table 12:** Breed by environment interaction on survivability of birds (male and female) during growing in ACGG Nigeria project zones (6–18 weeks) (2016–2017).

State	Breeds	IN	FN	Survival probability ± SE	Cumulative hazard ± SE	Log rank (p value)
Imo	Fulani	792	745	0.908 ± 0.011	0.097 ± 0.011	7.10 × 10${}^{-269}$
	FUNAAB alpha	739	625	0.842 ± 0.016	0.172 ± 0.016	
	Kuroiler	2100	1924	0.864 ± 0.009	0.146 ± 0.009	
	Noiler	1680	1371	0.754 ± 0.014	0.282 ± 0.014	
	Sasso	2520	2187	0.827 ± 0.009	0.19 ± 0.009	
	Shika-Brown	2520	2331	0.904 ± 0.006	0.101 ± 0.006	
Kebbi	Fulani	865	805	0.896 ± 0.012	0.11 ± 0.012	
	FUNAAB alpha	1195	1032	0.823 ± 0.013	0.194 ± 0.013	
	Kuroiler	2064	1823	0.829 ± 0.01	0.188 ± 0.01	
	Noiler	1664	1327	0.751 ± 0.014	0.286 ± 0.014	
	Sasso	2130	1878	0.823 ± 0.01	0.194 ± 0.01	
	Shika-Brown	2520	2200	0.843 ± 0.009	0.171 ± 0.009	
Kwara	Fulani	570	475	0.789 ± 0.022	0.236 ± 0.022	
	FUNAAB alpha	1230	1028	0.752 ± 0.016	0.285 ± 0.016	
	Kuroiler	2100	1592	0.71 ± 0.014	0.342 ± 0.014	
	Noiler	1659	1242	0.649 ± 0.018	0.432 ± 0.018	
	Sasso	2498	2016	0.747 ± 0.012	0.291 ± 0.012	
	Shika-Brown	2493	1900	0.694 ± 0.013	0.365 ± 0.013	
Nasarawa	Fulani	740	574	0.761 ± 0.021	0.273 ± 0.021	
	FUNAAB alpha	1188	1155	0.95 ± 0.007	0.051 ± 0.007	
	Kuroiler	1791	1330	0.728 ± 0.014	0.318 ± 0.014	
	Noiler	1820	1809	0.992 ± 0.002	0.008 ± 0.002	
	Sasso	2087	1580	0.74 ± 0.013	0.301 ± 0.013	
	Shika-Brown	2121	1951	0.898 ± 0.007	0.107 ± 0.007	
Rivers	Fulani	715	630	0.839 ± 0.016	0.175 ± 0.016	
	FUNAAB alpha	1262	1128	0.859 ± 0.011	0.152 ± 0.011	
	Kuroiler	1954	1552	0.719 ± 0.014	0.331 ± 0.014	
	Noiler	1506	1026	0.647 ± 0.019	0.435 ± 0.019	
	Sasso	2515	1967	0.728 ± 0.012	0.317 ± 0.012	
	Shika-Brown	2190	1883	0.8 ± 0.011	0.223 ± 0.011	

IN and FN: initial and final number of birds SE: standard error; log rank: test of homogeneity for differences in survival.

#### 3.7.2 Laying phase (20–72 weeks)

**Figure 16:**
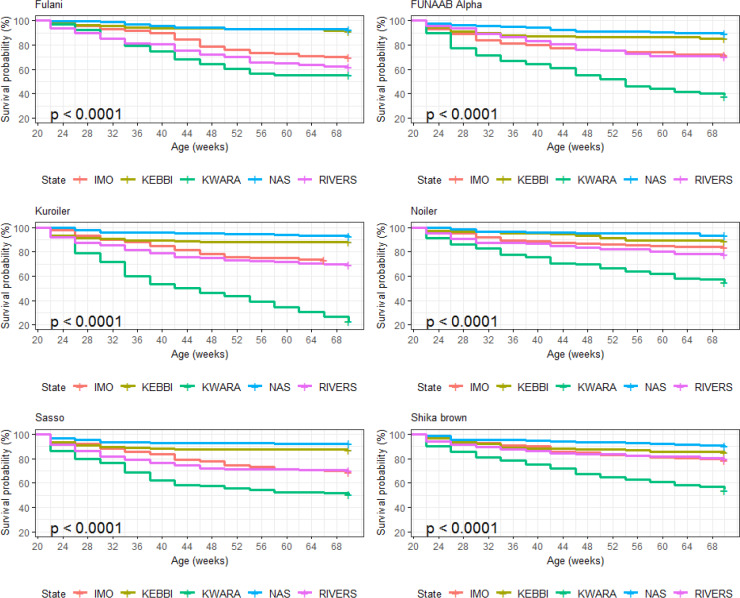
Breed by environment interaction on survivability of female birds (breeds) raised on-farm in ACGG Nigeria project zones (20–72 weeks) (2016–2018).

**Figure 17:**
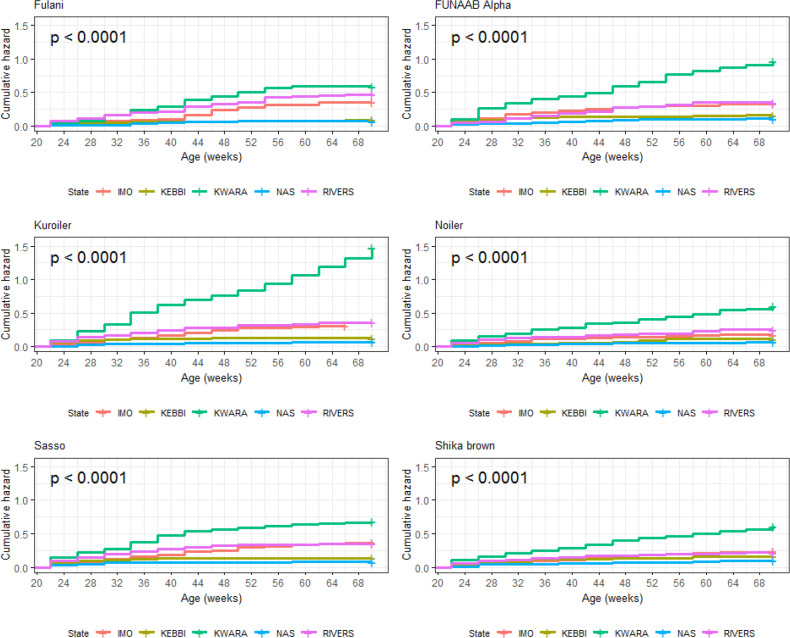
Breed by environment interaction on cumulative hazard of female birds (breeds) raised on-farm in ACGG Nigeria project zones (20–72 weeks) (2016–2018).

**Figure 18:**
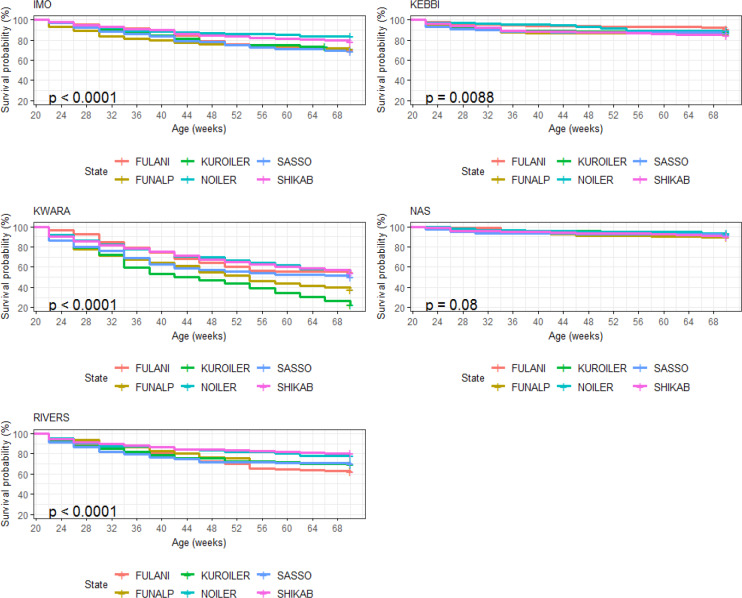
Breed by environment interaction on survivability of female birds (agroecologies) raised on-farm in ACGG Nigeria project zones (20–72 weeks) (2016–2018).

**Figure 19:**
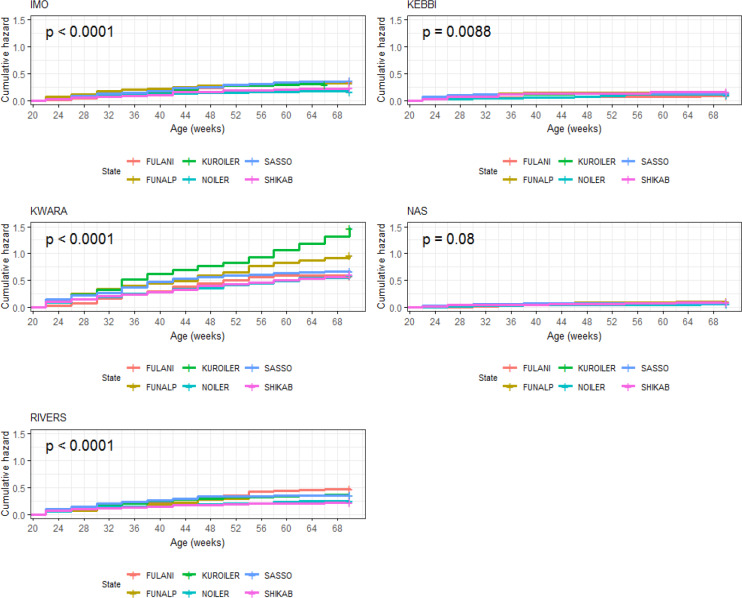
Breed by environment interaction on cumulative hazard of female birds raised on-farm in ACGG Nigeria project zones (22–70 weeks) (2016–2018).

Breed × environment interaction on survivability of birds was significant (p<0.0001) during the laying phase (Table 13). For the relative survival probabilities across agroecologies and breeds, Noiler (0.84) and Shika-Brown (0.79) ranked first and second, while the lowest-ranked genotype was Sasso (0.69) in Imo. Fulani (0.92) and Noiler (0.89) were in first and second position, while Shika-Brown (0.85) was ranked lowest in Kebbi. Fulani (0.55) and Noiler (0.55) were ranked first and second while Kuroiler (0.23) was the lowest in Kwara. In Nasarawa, the survivability potential was 0.94 (Noiler and FUNAAB Alpha), while in Rivers, Shika-Brown (0.80) and Fulani (0.62) had the highest and lowest survivability potential, respectively (Table 13). Overall, Kwara had the lowest survivability (Fig. 16), while Nasarawa had the highest survivability for all the breeds during laying. Kuroiler (1.48) had the highest cumulative hazard for probabilities of death in Kwara (Fig. 17). The agroecological zone effect on survival probability revealed that Nasarawa had the highest probabilities for all the breeds (Fig. 18). Kebbi ranked next in survival probability, followed by Imo; Rivers and Kwara were lowest in ranking. The cumulative hazard risk was the lowest for Fulani across all the five agroecologies (Fig. 19). The cumulative risk of death was highest in Kwara for all the six breeds, while Nasarawa had the lowest risk.

**Table 13:** Breed by environment interaction on survivability of female birds raised on-farm in ACGG Nigeria project zones (22–70 weeks) (2016–2018).

State	Breeds	IN	FN	Survival probability ± SE	Cumulative hazard ± SE	Log rank (p value)
Imo	Fulani	399	279	0.699 ± 0.033	0.358 ± 0.033	0
	FUNAAB alpha	331	238	0.716 ± 0.035	0.334 ± 0.035	
	Kuroiler	822	606	0.735 ± 0.021	0.308 ± 0.021	
	Noiler	607	510	0.84 ± 0.018	0.174 ± 0.018	
	Sasso	1210	845	0.69 ± 0.019	0.371 ± 0.019	
	Shika-Brown	1057	843	0.786 ± 0.016	0.241 ± 0.016	
Kebbi	Fulani	433	398	0.915 ± 0.015	0.089 ± 0.015	
	FUNAAB alpha	542	463	0.854 ± 0.018	0.158 ± 0.018	
	Kuroiler	900	794	0.882 ± 0.012	0.125 ± 0.012	
	Noiler	526	469	0.892 ± 0.015	0.115 ± 0.015	
	Sasso	945	825	0.873 ± 0.012	0.136 ± 0.012	
	Shika-Brown	971	829	0.85 ± 0.014	0.163 ± 0.014	
Kwara	Fulani	253	140	0.553 ± 0.056	0.592 ± 0.056	
	FUNAAB alpha	501	201	0.381 ± 0.057	0.964 ± 0.057	
	Kuroiler	638	171	0.229 ± 0.073	1.475 ± 0.073	
	Noiler	482	277	0.548 ± 0.041	0.602 ± 0.041	
	Sasso	960	493	0.506 ± 0.032	0.681 ± 0.032	
	Shika-Brown	978	557	0.542 ± 0.029	0.613 ± 0.029	
Nasarawa	Fulani	253	235	0.929 ± 0.017	0.074 ± 0.017	
	FUNAAB alpha	539	484	0.896 ± 0.015	0.11 ± 0.015	
	Kuroiler	512	478	0.926 ± 0.013	0.077 ± 0.013	
	Noiler	872	815	0.935 ± 0.009	0.068 ± 0.009	
	Sasso	685	633	0.924 ± 0.011	0.079 ± 0.011	
	Shika-Brown	840	763	0.905 ± 0.011	0.1 ± 0.011	
Rivers	Fulani	363	227	0.623 ± 0.041	0.474 ± 0.041	
	FUNAAB alpha	551	389	0.706 ± 0.027	0.348 ± 0.027	
	Kuroiler	663	463	0.697 ± 0.026	0.361 ± 0.026	
	Noiler	308	240	0.779 ± 0.03	0.249 ± 0.03	
	Sasso	1009	712	0.703 ± 0.02	0.353 ± 0.02	
	Shika-Brown	871	701	0.803 ± 0.017	0.22 ± 0.017	

IN and FN: initial and final number of birds; SE: standard error; log rank: test of homogeneity for differences in survival.

## 4 Discussion

### 4.1 Growth performance of six breeds of chicken

An on-farm study provides a more realistic performance of tested birds under farmers' management practices (Sorensen, 2010). Significant breed variations in growth performance of male and female birds of the six breeds tested on-farm in five agroecological zones were revealed. Male birds had a fast growth rate from 6 to 10 weeks and a slower growth rate from 14 to 18 weeks old. Noiler showed an unusually higher body weight gain between 10 and 14 weeks, which was different from the other breeds. Breed differences in productivity and survivability of Vanaraja, Rhode Island Red (RIR) and Deshi birds in the Gorkhaland Territorial Administration (GTA) – a semi-autonomous administrative body for the Darjeeling Hills in West Bengal, India – have been documented by Roy et al. (2017). The performance of Vanaraja, a dual-purpose breed, was better than RIR in terms of body weight gain from 4 to 20 weeks of age with reduced mortality. Noiler, also a dual-purpose breed developed in Nigeria, showed better performance in body weight gain than Kuroiler and Sasso, which are also dual-purpose and tropically adapted breeds but not indigenous to Nigeria.

Compared with the average male body weight (680 g) of local chickens at 18 weeks (Nwosu, 1979; Nwosu and Asuquo, 1985; Olori and Sonaiya, 1992; Adedokun and Sonaiya, 2002; Ajayi, 2010) the breeds were higher by 119.7 % (Fulani), 143.9 % (Shika-Brown), 176.9 % (FUNAAB Alpha), 204.5 % (Kuroiler), 205.6 % (Sasso) and 214.9 % (Noiler). This shows the clustering of the breeds into two groups of faster-growing (Kuroiler, Sasso and Noiler), and slower-growing breeds (Fulani, FUNAAB Alpha and Shika-Brown).

### 4.2 Effect of agroecologies on growth performance of birds

On-farm trials revealed that agroecologies had a significant effect on the live body weight of the six breeds studied. Hassan et al. (2018) earlier reported that there was a breed × agroecology interaction effect on the body weight of these six breeds at the brooding stage (0–6 weeks). The difference in the environmental factors across the five agroecologies was adjusted by the CV for each variable. Growth performance of female birds during the laying period was affected by agroecology. Laying birds have been reported to differ in their adaptability to husbandry systems (Yakubu et al., 2007) and climatic factors (Garcês et al., 2001). An increase in body weight during the laying period as was observed in Kebbi (Sudan savanna) was at variance with the reports of Garcês et al. (2001) that elevated temperatures reduced the body weight of laying birds.

### 4.3 Breed × agroecology interaction effect on egg production

A higher total number of birds at 72 weeks in Kebbi (Sudan savanna/northern Guinea savanna) did not correspond to higher HDEP; rather, birds in Imo (lowland rainforest and freshwater swamp) had higher HDEP than those in Kebbi. Hot dry agroecologies have been reported to reduce egg number (Garcês et al., 2001) and increase the probability of death (Shittu et al., 2014) in laying birds. High HDEP in Imo (62.84 %) and Rivers (57.40 %), lowland rainforest and freshwater swamp, respectively, may be attributed to lower ambient temperatures in the two zones compared with higher ambient temperatures in Kwara (23.18 %), Nasarawa (33.50 %) and Kebbi (41.36 %). The HDEP observed in this study was higher (Imo and Rivers) and lower (Kwara, Nasarawa, Kebbi) than the 44.7 % (rainforest), 53.5 % (Guinea savanna) and 54.9 % (derived savanna) previously reported by Adedokun and Sonaiya (2001) for local chickens collected from those agroecologies and raised intensively. Birds in this study were raised under the semi-scavenging system of production. The difference in the two results could be due to the different management systems adopted. The semi-scavenging/semi-intensive systems, in which feed quality and quantity are subject to farmers' ability to provide supplementary feed and the amount of scavengeable feed resource (SFRs) available (Sonaiya, 2004), may explain some of the variations in the HDEP observed in this study. Jacob et al. (2017) have asserted that egg production in backyard chicken flocks is affected by management and environmental factors, especially temperature, sometimes causing a sudden drop in egg production. During the laying phase the six chicken breeds also maintained a relatively uniform weight as revealed by the lower CV recorded at this period than what obtains in the growing phase. Shika-Brown had the highest HDEP. This was expected as Shika-Brown is more of an egg-type genotype than dual-purpose. FUNAAB Alpha ranked second in HDEP. Egg number had previously been reported as one of the significant traits influencing farmers' breed preference (Yakubu et al., 2019). The average egg weight of the six breeds of chicken was higher by 146 % compared to the 35 g reported for the local eggs (Adedokun and Sonaiya, 2002; Ajayi, 2010).

### 4.4 Survival probability and hazard risk factors associated with birds

Actual mortality did not include birds sold or consumed by the household or lost to predators. The overall mortality rate during growing and laying phases was highest in Kwara (derived savanna) and lowest in Imo (lowland forest). Tadesse (2014) reported higher mortality and lower survival of chicks in lowland than in midland agroecologies in northern Ethiopia.

The high mortality rate recorded between 6 and 18 weeks of age coincided with the period of peak rainfall that favours the spread of various disease pathogens in the tropics. Average daily temperature and relative humidity ranged from 26.4 ${}^{\circ }$C (Imo) to 28.4 ${}^{\circ }$C (Nasarawa) and 74.0 % (Nasarawa) to 80.0 % (Imo), respectively. Talukder et al. (2010) reported that high temperature and high humidity may negatively affect the growth and physiology of birds. Compared with Kuroiler, Noiler and Sasso, the higher survivability of FUNAAB Alpha, Fulani and Shika-Brown may be attributed to their adaptability to the prevailing environmental conditions (Yakubu and Ari, 2018). Indigenous chickens possess higher natural antibodies that aid their survival (Wondmeneh et al., 2015) and adaptability (Sankhyan and Thakur, 2018) in the extensive production system.

Fulani, an indigenous strain commonly found within the kraals of nomadic Fulanis, showed the highest survivability in all the five agroecologies. A higher probability of mortality for Kuroiler, Sasso and Noiler in the growing phase could be indicative of the need for good management of the birds to minimize stressful conditions in the early growing phase. According to Shittu et al. (2014), hot dry seasons that coincide with the months of February to May have been indicated for a spike in mortality with reduced egg production in laying hens raised in northwest Nigeria.

## 5 Conclusion

The results from this study showed that all the breeds had superior growth and laying performance compared to the local chickens. The group of Kuroiler, Sasso and Noiler had higher male body weight compared to FUNAAB Alpha, Shika-Brown and Fulani. The HDEP for Shika-Brown, FUNAAB Alpha and Kuroiler was higher than for Fulani, Noiler and Sasso, while Kuroiler and Sasso had higher egg weights. Ranking of the breeds (from highest to lowest) in terms of growth, laying performance and survivability was as follows: Shika-Brown/Sasso, FUNAAB Alpha/Noiler, Kuroiler and Fulani. The agroecological zones most suitable for the production and performance of the breeds, under the backyard scavenging management system, were ranked (from highest to lowest) as follows: wet lowland rainforest and freshwater swamp (Imo State), Sudan and northern Guinea savanna (Kebbi State), derived and southern Guinea savanna (Nasarawa State), mangrove swamp and freshwater swamp (Rivers State), and southern Guinea and northern Guinea savanna (Kwara State). The findings from this study show the potential of improved, dual-purpose breeds for increased smallholder poultry production.

### Ethics approval and consent to participate

The ethical guidelines were approved by International Livestock Research Institute (ILRL-IREC2015-08/1).

## Data Availability

All raw data are available as open access at (Dessie et al., 2017).
